# Highly infectious prions are not directly neurotoxic

**DOI:** 10.1073/pnas.2007406117

**Published:** 2020-09-08

**Authors:** Iryna Benilova, Madeleine Reilly, Cassandra Terry, Adam Wenborn, Christian Schmidt, Aline T. Marinho, Emmanuel Risse, Huda Al-Doujaily, Michael Wiggins De Oliveira, Malin K. Sandberg, Jonathan D. F. Wadsworth, Parmjit S. Jat, John Collinge

**Affiliations:** ^a^Medical Research Council Prion Unit at University College London (UCL), UCL Institute of Prion Diseases, London W1W 7FF, United Kingdom

**Keywords:** prions, neurotoxicity, neurodegeneration

## Abstract

Prions are infectious agents which cause rapidly lethal neurodegenerative diseases in humans and animals following long, clinically silent incubation periods. They are composed of multichain assemblies of misfolded cellular prion protein. While it has long been assumed that prions are themselves neurotoxic, recent development of methods to obtain exceptionally pure prions from mouse brain with maintained strain characteristics, and in which defined structures—paired rod-like double helical fibers—can be definitively correlated with infectivity, allowed a direct test of this assertion. Here we report that while brain homogenates from symptomatic prion-infected mice are highly toxic to cultured neurons, exceptionally pure intact high-titer infectious prions are not directly neurotoxic. We further show that treatment of brain homogenates from prion-infected mice with sodium lauroylsarcosine destroys toxicity without diminishing infectivity. This is consistent with models in which prion propagation and toxicity can be mechanistically uncoupled.

## Significance

Prions are infectious agents composed of polymers of misfolded prion protein which cause fatal brain diseases such as Creutzfeldt–Jakob disease. These diseases involve progressive loss of neuronal cells, and it has been long assumed that prions are directly toxic to cells as they propagate. However, recent studies have suggested that prion infectivity and neurotoxicity can be uncoupled and involve distinct mechanisms. Using highly purified infectious prions we demonstrate that prions are not directly neurotoxic and that toxicity present in infected brain tissue can be distinguished from infectious prions. This has fundamental implications for understanding of prion diseases and how to treat them and may have wide relevance in other neurodegenerative diseases which involve propagation and spread of proteopathic seeds.

Prion diseases are fatal transmissible neurodegenerative conditions affecting humans and a range of other mammalian species (1, 2). Infectious prions are composed of multichain assemblies of misfolded host-encoded cellular prion protein (PrP^C^) which propagate by recruitment of further PrP^C^ and subsequent fission (1, 3–5). Multiple studies have demonstrated neurotoxicity of crude or semipurified preparations of brain homogenate from prion-infected animals in cultured primary mouse neurons or mouse brain slices in vitro (6–10). However, prion infections are associated with accumulation of a diverse range of PrP assemblies, only a minority of which meet the biochemical definition (with characteristic resistance to proteinase K [PK] digestion) of PrP^Sc^, a term often used synonymously with prion infectivity (1, 11–14). We have previously demonstrated that, following an initial phase of exponential prion propagation in vivo, which is not rate-limited by PrP^C^ expression level, infective titer reaches a plateau level, and the duration of this plateau phase to clinical onset is inversely proportional to PrP^C^ expression level (15). During this plateau phase there is accumulation of protease-sensitive disease-related forms of PrP at a rate linearly proportional to the PrP^C^ expression level (14). It is during this second phase that neuropathology becomes established. We propose that a toxic PrP species, PrP^L^, is generated during this phase with clinical onset occurring when PrP^L^ reaches a neurotoxic threshold (3, 14). This model proposes that infectious prions are themselves not toxic but that a pathway switch to production of PrP^L^ once a plateau level of infectivity is reached is responsible for the synaptotoxicity and neurodegeneration that leads to clinical onset (3, 14, 15). This model is also supported by the recognition of subclinical prion infections where animals may live a normal life span despite harboring high prion titers in their brains (16–19).

A major obstacle to defining the neurotoxic PrP species has been the development of a suitable in vitro assay with sufficient dynamic range that will readily discriminate between normal and prion-infected brain. Here we report a multiparametric assay of neuronal morphology and show that neurite length, neurite fragmentation, and dendritic spine density unequivocally differentiate prion-infected from noninfected mouse brain toxicity. Remarkably, highly purified mouse prions (20–22) show no toxic response in this assay. Moreover, we show that treatment of brain homogenates from prion-infected mice with sodium lauroylsarcosine (≥2% [wt/vol] final concentration) destroys toxicity without reducing the prion infectivity titer, thereby clearly demonstrating that infectious prion assemblies can be uncoupled from neurotoxicity. The advances reported here will facilitate isolation of the neurotoxic PrP species and its structural characterization.

## Results

### Purified Rocky Mountain Laboratory Prions Are Not Neurotoxic in Cell Culture.

We isolated prions from the brains of clinically sick mice that had been intracerebrally inoculated with the Rocky Mountain Laboratory (RML) prion strain using an established method to isolate exceptionally pure high-titer prions (20). This method maintains the strain properties of RML prions, and the specific infectivity of such purified RML prions (pRML) was accurately determined by Automated Scrapie Cell Assay (ASCA) (23). The infectious titer of the pRML stock solutions prepared in Dulbecco’s phosphate-buffered saline (D-PBS) was 10^7.9^^±^^0.2^ infectious units (IU)/mL with a specific prion infectivity of 10^9.5^^±^^0.2^ IU/mg protein.

**Fig. 1.**
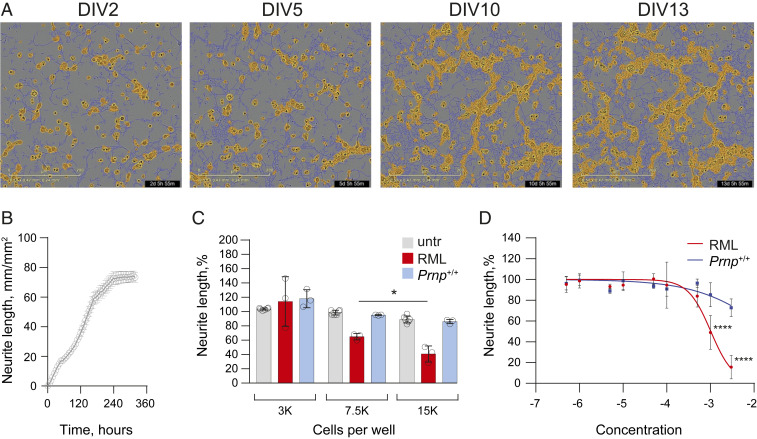
Development of the IncuCyte-based neurite retraction assay. (*A* and *B*) Neurite outgrowth in primary neurons plated at 15,000 cells/well plateaued after 10 d in vitro (DIV); *n* = 6. Dark blue traces: neurites; yellow: cell features excluded from neurite analysis. (Scale bar, 200 µm.) (*C*) Neurite retraction in response to the treatment with RML-infected and uninfected *Prnp*^+/+^ brain diluted to a concentration of 10^−3^ (RML, 10^5.2^ IU/mL) for 12 h occurred in a cell-density–dependent manner (*n* = 3, *P < 0.03, unpaired two-tailed *t* test). (*D*) Dose–response curve for the neurite retraction assay; *n* = 3 to 12 independent cell cultures were analyzed in triplicate after 12 h of treatment; mean ± SD. Data were fitted using a nonlinear regression model **[**Y = 100/(1+10^((LogIC50-X)×HillSlope))**]**. ****P < 0.0001, one-way ANOVA with Tukey’s multiple comparison test.

**Fig. 2.**
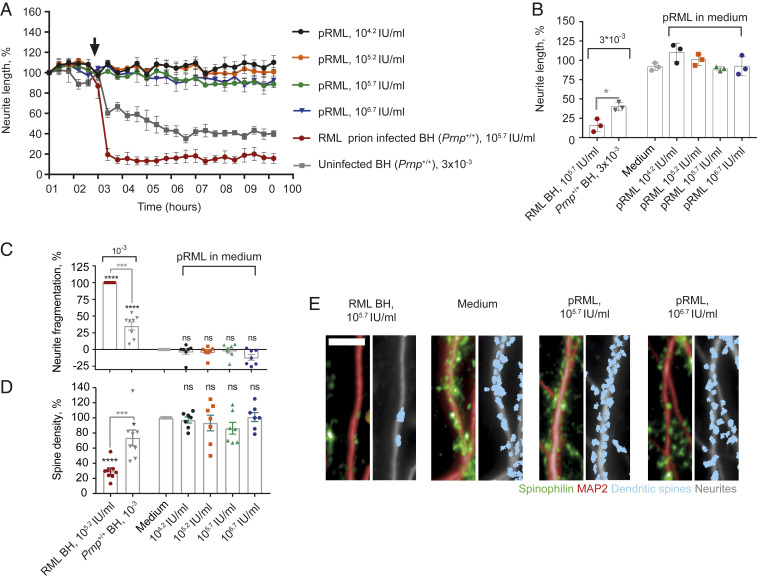
Purified RML prions are not neurotoxic in primary cell culture. (*A* and *B*) Neurite length after treatment (designated by arrow) with pRML diluted in tissue culture medium at 10^4.2^ (*n* = 3), 10^5.2^ (*n* = 3), 10^5.7^ (*n* = 3), and 10^6.7^ (*n* = 3) IU/mL or RML prion-infected brain (RML BH) at a concentration of 3 × 10^−3^ in medium (10^5.7^ IU/mL, *n* = 3) or uninfected brain at a concentration of 3 × 10^−3^ in medium (*n* = 3). (*A*) Normalized response over time, mean ± SEM, (*B*) at 72 h post treatment, mean ± SD; *P < 0.05 (unpaired two-tailed *t* test). (*C* and *D*) Analysis of neurite fragmentation (*C*) and dendritic spine density (*D*) after treatment with pRML in medium at 10^4.2^ (*n* = 7), 10^5.2^ (*n* = 7), 10^5.7^ (*n* = 7), 10^6.7^ IU/mL (*n* = 6), RML brain at a concentration of 10^−3^ in medium (10^5.2^ IU/mL, *n* = 8), uninfected brain at a concentration of 10^−3^ in medium (*n* = 7) or medium alone (*n* = 8), mean ± SEM, *P < 0.05, ***P < 0.001, ****P < 0.0001. Gray asterisks: comparison of RML BH to uninfected control BH, unpaired two-tailed *t* test. Black asterisks: comparison to medium only, one-way ANOVA with Sidak’s correction for multiple comparisons; ns: not significant. (*E*) Representative images of dendritic spine density; green and red: raw imaging data; blue and gray: image analysis. (Scale bar, 10 µm.)

**Fig. 3.**
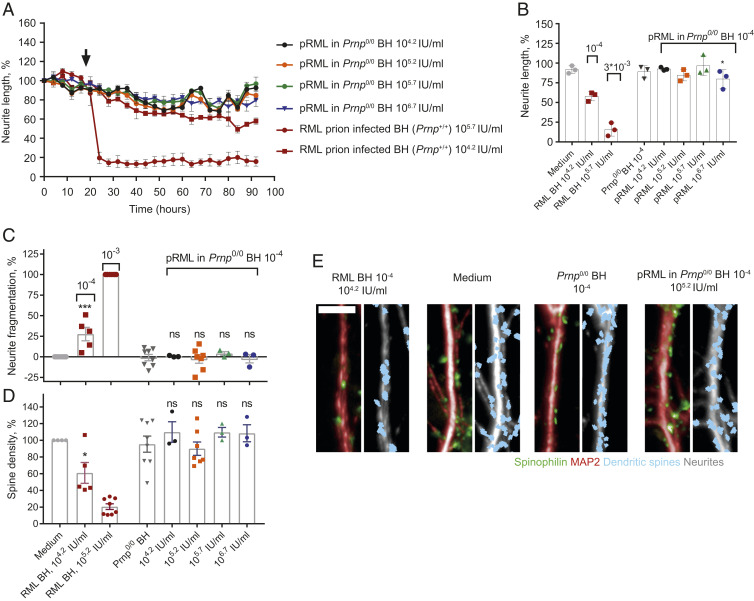
Purified RML prions in *Prnp*^*0/0*^ mouse brain homogenate are not neurotoxic in primary cell culture. (*A* and *B*) Neurite length after treatment (designated by arrow) with pRML in *Prnp*^*0/0*^ BH at a concentration of 10^−4^ at 10^4.2^ (*n* = 3), 10^5.2^ (*n* = 3), 10^5.7^ (*n* = 3), and 10^6.7^ IU/mL (*n* = 3) or RML BH at a concentration of 3 × 10^−3^ (10^5.7^ IU/mL, *n* = 3), 10^−4^ (10^4.2^ IU/mL, *n* = 3) or *Prnp*^*0/0*^ BH at 10^−4^ (*n* = 3). The same dataset for RML BH at concentrations of 3 × 10^−3^ and 10^−4^ is shown across the figures for comparison with pRML. (*A*) Normalized response over time, mean ± SEM (*B*) at 72 h post treatment and mean ± SD pRML in *Prnp*^*0/0*^ BH (10^−4^) at 10^6.7^ IU/mL is significantly less toxic than RML BH at 10^4.2^ IU/mL (10^−4^). *P < 0.05, unpaired two-tailed *t* test. (*C* and *D*) Analysis of neurite fragmentation (*C*) and dendritic spine density (*D*) after treatment with pRML in *Prnp*^*0/0*^ BH at a concentration of 10^−4^ at 10^4.2^ (*n* = 3), 10^5.2^ (*n* = 7), 10^5.7^ (*n* = 3), and 10^6.7^ IU/mL (*n* = 3) and *Prnp*^*0/0*^ BH at a concentration of 10^−4^ (*n* = 8), RML BH at concentration of 10^−4^ (10^4.2^ IU/mL, *n* = 5) and 10^−3^ (10^5.2^ IU/mL, *n* = 8), or medium alone (*n* = 8), mean ± SEM; comparison to *Prnp*^*0/0*^ BH at 10^−4^, one-way ANOVA with Sidak’s correction for multiple comparisons. *P < 0.05 and ***P < 0.001 ns: not significant. (*E*) Representative images of dendritic spine density; green and red: raw imaging data; blue and gray: image analysis. (Scale bar, 10 µm.)

**Fig. 4.**
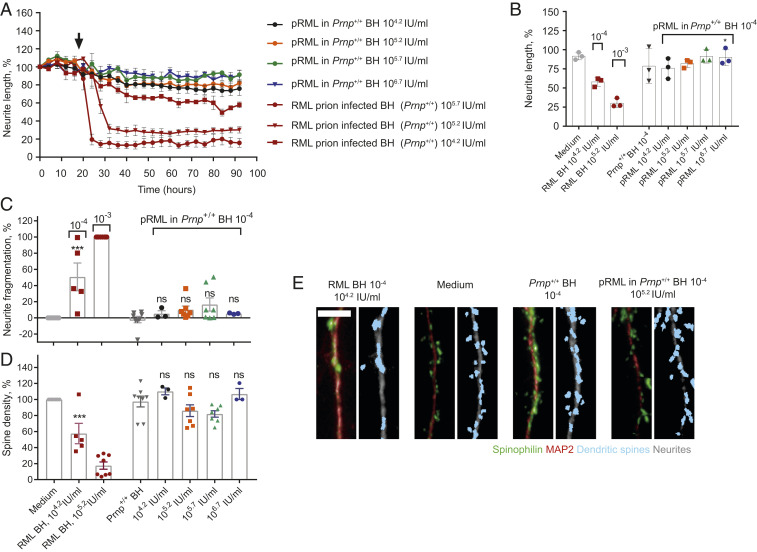
Purified RML prions in *Prnp*^*+/+*^ mouse brain homogenate are not neurotoxic in primary cell culture. (*A* and *B*) Neurite length after treatment (designated by arrow) with pRML in *Prnp*^*+/+*^ BH at a concentration of 10^−4^ at 10^4.2^ (*n* = 3), 10^5.2^ (*n* = 3), 10^5.7^ (*n* = 3), and 10^6.7^ IU/mL (*n* = 3), uninfected *Prnp*^*+/+*^ BH at a concentration of 10^−4^ (*n* = 3), or RML BH at concentrations of 10^−4^ (10^4.2^ IU/mL, *n* = 3), 10^−3^ (10^5.2^ IU/mL, *n* = 3), and 3 × 10^−3^ (10^5.7^ IU/mL, *n* = 3). The same dataset for RML BH at concentrations of 3 × 10^−3^ and 10^−4^ is shown across the figures for comparison with pRML. (*A*) Normalized response over time, mean ± SEM, (*B*) at 72 h post treatment; mean ± SD pRML in *Prnp*^*+/+*^ BH (10^−4^) at 10^6.7^ IU/mL is significantly less toxic than RML BH at 10^4.2^ IU/mL (10^−4^); *P < 0.05, unpaired two-tailed *t* test. (*C* and *D*) Analysis of neurite fragmentation (*C*) and dendritic spine density (*D*) after treatment with pRML in *Prnp*^*+/+*^ BH at a concentration of 10^−4^ at 10^4.2^ (*n* = 3), 10^5.2^ (*n* = 7), 10^5.7^ (*n* = 7), and 10^6.7^ IU/mL (*n* = 3) and *Prnp*^*+/+*^ BH at a concentration of 10^−4^ (*n* = 8), RML BH at a concentration of 10^−4^ (10^4.2^ IU/mL, *n* = 5) and 10^−3^ (10^5.2^ IU/mL, *n* = 8) or medium alone (*n* = 8); mean ± SEM, comparison to *Prnp*^*+/+*^ BH at 10^−4^, one-way ANOVA with Sidak’s correction for multiple comparisons; ***P < 0.001; ns: not significant. (*E*) Representative images of dendritic spine density. Green and red: raw imaging data; blue and gray: image analysis (Scale bar: 10 µm.)

We assessed neurotoxicity of pRML in a high-throughput, multiparametric assay consisting of time-course studies and end-point analyses of neuronal morphology. Neurite retraction was measured in phase-contrast mode in a cortico-hippocampal mouse neuronal culture on an IncuCyteS3 live-cell imaging platform followed by immunocytochemistry and high-content image analysis of neurons on an Opera Phenix platform. For immunostaining we used the neuron-specific markers NeuN, MAP2, and spinophilin that detected neuronal nuclei, neurites, and dendritic spines, respectively. We first examined toxicity of RML prion-infected and normal, uninfected, brain homogenate in these assays. Brain homogenate dilutions from clinically sick RML prion-infected wild type (*Prnp*^+/+^) mice containing from 10^4.2^ to 10^5.7^ IU/mL showed marked neurotoxicity (Figs. 1–4 and Movies S1 and S2) and, although normal brain homogenate at higher concentrations also showed nonspecific toxic effects, this was never comparable to the extent of toxicity elicited by RML-infected brain homogenate. Indeed, when applied over a range of equivalent concentrations, RML-infected brain homogenate was always significantly more toxic than normal brain homogenate (Figs. 1–4 and *SI Appendix*, Fig. S2, and Movies S1 and S2). Importantly, no significant prion propagation was detected by ASCA in primary mouse neurons treated with infectious RML brain homogenates for 72 h (*SI Appendix*, Fig. S1), indicating that prion propagation was not the basis for the observed specific neurotoxicity. In sharp contrast to findings with RML-infected brain homogenate, pRML prions, diluted in tissue culture medium, did not induce neurite retraction even when as much as 10^6.7^ IU/mL was added, equivalent to a 10-fold higher prion titer than that present in the brain homogenate eliciting maximal toxicity (Fig. 2 *A* and *B*). We also evaluated neurite fragmentation (Fig. 2*C*) and dendritic spine density (Fig. 2 *D* and *E*) and found that none of the pRML preparations induced toxicity in the cultured neurons at any stage of treatment, again in sharp contrast to RML prion-infected brain homogenate (Fig. 2 and Movie S3).

### Purified RML Prions in *Prnp*^*0/0*^ Mouse Brain Homogenate Are Not Neurotoxic in Cell Culture.

It is possible that, in order to cause neurotoxicity, infectious prions require cofactors present in mouse brain. Therefore we resuspended pRML prions in brain homogenate from uninfected *Prnp*^*0/0*^ mice (which are devoid of PrP^C^ and unable to propagate prions) (24, 25). We tested four concentrations of infectious pRML containing 10^4.2^ to 10^6.7^ IU/mL using the above-described multiparametric assay of neurotoxicity. As with pRML prions in tissue culture media, pRML prions diluted in *Prnp*^*0/0*^ brain homogenate prior to application to neurons showed no evidence of neurotoxicity as measured by neurite retraction (Fig. 3 *A* and *B*, and Movie S4), neurite fragmentation (Fig. 3*C*), or reduction in spine density (Fig. 3 *D* and *E*). *Prnp*^*0/0*^ brain homogenate itself at a concentration of 10^−4^ did not cause toxicity in primary neurons, whereas RML prion-infected brain at the same concentration was toxic (Fig. 3 *B*–*E*, and Movies S1 and S5).

### Purified RML Prions in *Prnp*^*+/+*^ Mouse Brain Homogenate Are Not Neurotoxic in Cell Culture.

We also added pRML to brain homogenate from uninfected wild-type *Prnp*^*+/+*^ mice to levels of 10^4.2^ to 10^6.7^ IU/mL and applied it to primary neurons for 3 d as before. As incubation of pRML prions with *Prnp*^*+/+*^ brain homogenate could conceivably result in generation of prions or putative toxic PrP species in vitro prior to addition to the neuronal culture, we kept incubation time to a minimum and did not exceed 10 min at 37 °C. Normal brain homogenate at 10^−4^ concentration was selected as the vehicle for these spiking experiments because at this concentration there is minimal nonspecific toxicity, and the dynamic sensitivity of the assay to report potential toxicity of pRML is maximal (Figs. 3 and 4, and Movies S1, S2, and S4–S6). Four concentrations of pRML diluted in *Prnp*^*+/+*^ brain homogenate were tested. We did not observe neurite retraction even at the highest titer of infectious pRML (10^6.7^ IU/mL) in *Prnp*^*+/+*^ brain homogenate, equivalent to a 10-fold higher prion titer than that present in the brain homogenate eliciting maximal toxicity (Figs. 4*A* and 2*B* and Movie S6). Furthermore, no neurite fragmentation or dendritic spine loss was observed (Fig. 4 *C*–*E*). In sharp contrast, 10% (wt/vol) RML prion-infected brain homogenate diluted to 10^4.2^ IU/mL caused marked neurite fragmentation and spine loss (Fig. 4 *C* and *D*). Further experiments using a higher concentration of normal brain homogenate as vehicle for pRML spiking experiments are shown in *SI Appendix*, Fig. S2. While nonspecific toxicity from normal brain at 10^−3^ concentration was apparent, no additional toxicity was elicited by inclusion of high concentrations of pRML. Collectively, these data demonstrate that authentic ex vivo prions are not directly neurotoxic, either when directly added to cell cultures or when first mixed with uninfected brain homogenate to produce a prion titer at or far exceeding the prion titers of brain homogenate dilutions prepared from clinically sick RML prion-infected mice which are highly neurotoxic.

### Sarkosyl Abolishes the Toxicity of RML-Infected Brain Homogenate without Diminishing Infectious Prion Titer.

**Fig. 5.**
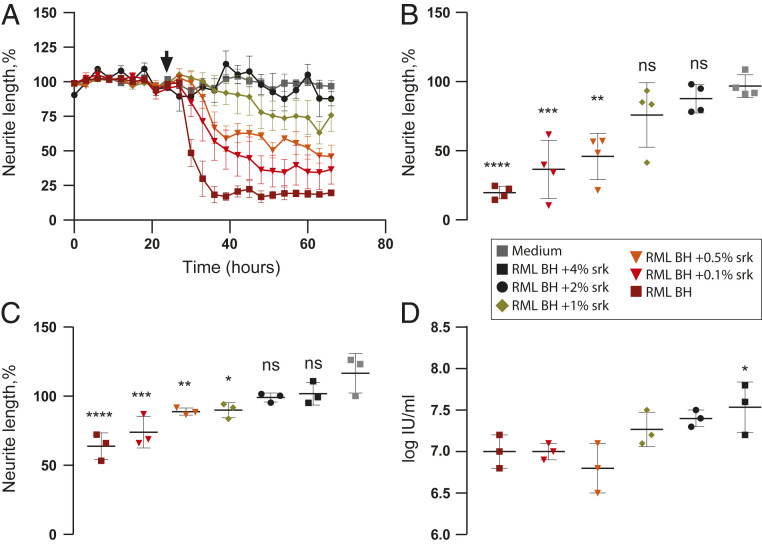
Toxicity of RML brain homogenate is abolished by increasing concentrations of sarkosyl while prion infectivity titer is fully preserved. RML BH (10% [wt/vol]) was adjusted to final concentrations of 0.1 to 4% (wt/vol) sarkosyl (srk) in the sample, incubated at 37 °C for 30 min, and analyzed for acute toxicity to primary neurons at a final brain concentration of 10^−3.1^ (*A* and *B*) and 10^−4.1^ (*C*). Infectivity of the sarkosyl-treated RML BH samples (*C*) was assessed simultaneously by using the ASCA (*D*). (*A* and *B*) Neurite length after treatment (designated by arrow) with RML BH at a concentration of 10^−3.1^ (*n* = 4). (*A*) Normalized response over time (*B*) at 56 h post treatment; mean ± SD, **P < 0.01, ***P < 0.001, and ****P < 0.0001, comparison to medium, one-way ANOVA with Sidak’s correction for multiple comparisons. ns: not significant. (*C*) Normalized response at 72 h post treatment (*n* = 3); mean ± SD, *P < 0.05, **P < 0.01, ***P < 0.001, ****P < 0.0001, comparison to medium, one-way ANOVA with Dunnett’s multiple comparisons test. ns: not significant. (*D*) Infectivity titer of RML BH treated with sarkosyl (*n* = 3) was determined by ASCA in PK1/2 cells after the third split. *P < 0.05, comparison to RML BH, one-way ANOVA with Dunnett’s multiple comparisons test.

In a further series of experiments, and as a prerequisite for undertaking fractionation of the toxic species from RML-infected brain homogenate, we sought to identify a detergent that is compatible with preservation of toxic activity. Based on the knowledge that sarkosyl has no detrimental effects on prion infectivity (20) we examined this detergent first. Remarkably, we found that treatment of 10% (wt/vol) RML brain homogenate with final concentrations of ≥2% (wt/vol) sarkosyl completely abolished toxicity in primary cultured neurons (Fig. 5 *A*–*C* and *SI Appendix*, Fig. S3) without diminishing the prion infectivity titer in the same samples as measured by the ASCA (Fig. 5*D*). Indeed, in accordance with previous findings (20), prion infectivity levels in the RML brain homogenates treated with the highest concentrations of sarkosyl were slightly higher than that of untreated RML brain homogenate which might reflect changes in infectious PrP rod aggregate size and number (21) or increased bioavailability of prions following membrane solubilization. In contrast to findings with RML brain homogenate, sarkosyl treatment had no apparent effect on the nonspecific toxic effects seen with high concentrations of normal brain homogenate (*SI Appendix*, Fig. S3). Thus, while the specific toxicity of RML brain homogenate is sarkosyl-sensitive, the nonspecific toxicity of normal brain homogenate is not. Indeed, after sarkosyl treatment the residual toxicity of RML brain homogenate becomes equivalent to that produced by the same concentration of normal brain homogenate (*SI Appendix*, Fig. S3). Collectively, these data with sarkosyl clearly suggest that the structure of the specific neurotoxic active species generated in RML-infected brain is distinct from that of infectious prions.

## Discussion

The molecular basis of prion neurotoxicity and neurodegeneration remains unknown. Here we report a multiparametric assay of prion neurotoxicity in primary neurons that readily differentiates prion-infected and noninfected mouse brain homogenates. This assay is a combination of kinetic and end-point high-content imaging of neuronal morphology, and it measures neurite retraction, neurite fragmentation, and changes in dendritic spine density upon semiacute treatment of 10-d-old cortico-hippocampal neuronal cultures in a multiwell plate. The 2 log_10_ dynamic range of the assay enabled us to undertake a systematic and quantitative investigation of prion toxicity in diseased mouse brains.

Using this multiparametric assay, we examined exceptionally pure preparations of highly infectious, ex vivo prion rods [100 to 200 nm in length (21, 22)] devoid of contaminating proteins (20) or small oligomeric PrP assemblies (21, 22) [akin to those described by Prusiner and colleagues (1, 26)] in cell culture alongside RML prion-infected mouse brain homogenates. We found that prion-infected brain homogenates were toxic as measured by neurite retraction and fragmentation as well as by reduction in dendritic spine density. In sharp contrast, highly purified infectious prion rods were not toxic and did not induce neurite retraction even when at a 10-fold higher prion titer than that present in the infected brain homogenate eliciting maximal toxicity. While highly purified infectious RML rods correspond to PK-resistant material, RML-infected brain homogenate (BH) comprises both PK-resistant and PK-sensitive prion species (12–14, 20). We found that a 30-min treatment of RML BH with increasing concentrations of sarkosyl abolished neurotoxicity while infectivity was unaffected or even slightly increased. Therefore, neurotoxicity does not seem attributable to infectious prion assemblies regardless of their aggregate size or relative protease sensitivity.

We do not consider these findings as contradictory to earlier publications which have reported neurotoxicity in partially purified prion preparations or crude prion-infected brain homogenate (7, 8), which contains multiple PrP assemblies of different sizes and relative protease resistance, making it impossible to attribute toxicity to a defined PrP species, infectious or otherwise. The lack of detectable direct toxicity of highly purified prion preparations or sarkosyl-treated infected brain homogenate is consistent with models of prion neurotoxicity being mediated by PrP species (PrP^L^), which are distinct from fibrillar infectious prion assemblies and which may be generated by a distinct mechanistic process (3, 4, 14, 15) although further studies are required to confirm the identity of neurotoxic species. In future work, these neurotoxicity assays will be applied to study the kinetics of the generation of neurotoxic species during the incubation period of prion infection and the biochemical properties of neurotoxic species, which should facilitate fractionation of prion-infected brain homogenate to isolate the neurotoxic species and allow their identification and structural characterization.

## Materials and Methods

All experimental procedures involving RML prions were carried out in microbiological containment level 2 or level 3 facilities with strict adherence to safety protocols and guidelines. Work with animals was performed in accordance with licenses approved and granted by the UK Home Office (Project Licenses 70/6454, 70/7274, and 70/9022) and conformed to University College London institutional and Animal Research: Reporting of In Vivo Experiments guidelines.

### Prion Inoculation and Brain Homogenate Preparation.

Mouse-adapted RML prions used in this study were originally supplied by Charles Weissmann, Zurich Institute for Molecular Biology. Two batches of 200 brains from terminally affected intracerebrally inoculated CD1 mice (Hsd:ICR [CD1]; Harlan) were homogenized in D-PBS lacking Ca^2+^ or Mg^2+^ ions (Invitrogen) using tissue grinders as described previously (20) to produce two ∼1-L pools of 10% (wt/vol) RML brain homogenate (designated I13100 and I17700). Control 10% (wt/vol) BH was prepared in D-PBS from the brains of uninfected CD1 (*Prnp*^+/+^) and FVB *Prnp* ablate (*Prnp*^0/0^) mice. All brain homogenates were stored as aliquots at −70 °C.

### Purification of Prion Rods from Brain Homogenate.

Ex vivo infectious RML prion rods were purified from 200-µL aliquots of 10% (wt/vol) of the RML brain homogenates I13100 and I17700 without the use of Proteinase K as described previously (20). The method produces a recovery of ∼10% of the prions present in the starting 10% (wt/vol) brain homogenate so that resuspension of the purified P4 pellet fraction in buffer at one-tenth of the volume of the 10% (wt/vol) brain homogenate from which it was derived produces prion preparations the infectivity titer of which is closely similar to that of the starting 10% (wt/vol) brain homogenate (20). Concentration of purified prions or buffer exchange was achieved by centrifugation at 16,100 × *g* for 30 min and resuspension of the pellet fraction into the desired volume and buffer of choice. Typically, we prepared stock preparations of the P4 pellet fraction resuspended in D-PBS containing 0.1% (wt/vol) sarkosyl in one-fiftieth of the volume of 10% (wt/vol) brain homogenate from which it was derived.

### ASCA of RML Infectivity.

The infectivity of purified RML prions and RML brain homogenate was determined by ASCA in RML prion-susceptible PK1/2 cells (27) as described (23). In brief, PK1/2 cells were dispensed into 96-well plates at 18,000 cells per well. The next day the cells were infected with prion-containing or mock samples using a Biomek FX liquid handling robot. After 3 d of infection, cells were split 1:8 by Biomek FX and grown to confluence for the next 3 d. After two further 1:8 splits, ∼25,000 cells were transferred into each well of activated ELISPOT plates, which were then treated with Proteinase K and developed using anti-PrP monoclonal antibody ICSM18 (D-Gen) and an alkaline-phosphatase–conjugated anti-IgG1 polyclonal secondary antibody (Southern Biotechnology Associates) as described (23). Spot counts reporting PK-resistant PrP-positive cells were determined with a Bioreader 5000-Eβ system (BioSys).

### Quantification of Prion Titer.

Enzyme-linked immunosorbent assay detection of protease-resistant PrP on prion-infected cells on ELISPOT plates results in the appearance of focal PrP “spots” distinct from any nonspecific background immunoreactivity seen on the plate (23, 27). The dose–response is dynamic between ∼50 and 1,000 spots per well; however, because the assay is nonlinear, every experiment must include serial dilution of RML prions of known prion titer (intracerebral LD_50_ units/mL determined from rodent bioassay) to produce a standard curve against which unknown samples can be calibrated. This method reports prion titer in tissue culture infectious units per milliliter, which in this paper for simplicity is abbreviated as “IU/mL.” A stock 10% (wt/vol) RML brain homogenate I8700 used as a reference preparation reported a prion titer of 10^7.2^ intracerebral LD_50_ units/mL when end-point–titrated once in Tg20 mice (20). The infectious titer of the 10% (wt/vol) RML BH stock (I13100) used in toxicity assays and for prion purification was measured in ASCA as described above and was 10^7.2^ IU/mL, consistent with previously determined titers of RML BH (14, 20). The infectious titer of four independently purified batches of prion rods (P4 pellets resuspended in D-PBS containing 0.1% [wt/vol] sarkosyl in one-fiftieth of the total volume of 10% [wt/vol] brain homogenate from which the pellets were derived) was evaluated in an ASCA in PK1/2 cells (as described above) and was equal to 10^7.9^^±^^0.2^ IU/mL and a specific prion infectivity of 10^9.5^^±^^0.2^ IU/mg protein, consistent with previous findings (20).

### Working Concentrations of Control and Prion-Infected Brain.

RML and uninfected brain dilutions were prepared from 10% (wt/vol) stock homogenates. As an example, a 10^−3^ concentration of brain was made by adding 10 µL of 10% (wt/vol) BH to 990-µL cell culture medium (a 10^−3^ concentration of brain in tissue culture medium therefore corresponds to 100-fold dilution of 10% [wt/vol] BH). As described above, the 10% (wt/vol) RML BH stock (I13100) used in toxicity assays and for prion purification has an infectivity titer of 10^7.2^ IU/mL or 10^8.2^ IU/g brain. To facilitate easy correlation, the values of RML brain concentration and corresponding infectivity titer (IU/mL) are provided here: 10^−1^ (10^7.2^), 10^−2^ (10^6.2^), 3 × 10^−3^ (10^5.7^), 10^−3^ (10^5.2^), 10^−4^ (10^4.2^), 10^−5^ (10^3.2^), 10^−6^ (10^2.2^), and 10^−7^ (10^1.2^).

### Detergent Treatment of Brain Homogenates.

Sarkosyl (20% [wt/vol, pH 7.7]) was prepared from *N*-lauroylsarcosine (sarkosyl) sodium salt (Sigma, catalog no. 61743) in sterile high-purity water. RML BH (10% [wt/vol]) or normal BH (10% [wt/vol]) were adjusted to final concentrations of 0.1 to 4% (wt/vol) sarkosyl in the sample and incubated at 37 °C with shaking at 300 × *g* in a thermomixer for 30 min. Subsequently, samples were diluted in cell culture medium and immediately analyzed for infectious titer in the ASCA in PK1/2 cells and for toxicity in primary neurons. The highest concentration of detergent in brain homogenate applied to cultured neurons was 0.04% (wt/vol) sarkosyl which had no intrinsic toxic effect on neuronal cells.

### Primary neuronal culture.

All of the reagents for primary neuron culture were from Thermo Fisher Scientific unless indicated otherwise. Primary cortico-hippocampal cultures for prion toxicity studies were prepared from embryonic day 17 FVB mouse brains. Cortices and hippocampi were dissected in chilled dissection medium consisting of calcium- and magnesium-free Hanks’s Balanced Salt Solution, 10 mM 4-(2-hydroxyethyl)-1-piperazineethanesulfonic acid, 2 mM L-glutamine and 100 U/mL of penicillin–streptomycin. Brain tissue was next treated with 0.25% (wt/vol) trypsin and 1,000 U of benzonase (VWR) for 15 min at 37 °C and washed three times with dissection medium. Cells were then resuspended in the plating medium consisting of Dulbecco’s Modified Eagle’s Medium supplemented with 10% (vol/vol) of heat-inactivated horse serum and 20 U/mL of penicillin–streptomycin. Next, the cells were triturated with fire-polished glass Pasteur pipettes with two different sizes of opening, filtered through 70-µm strainers, counted using a disposable Neubauer hemocytometer (Labtech), and plated out at 1.5 × 10^4^ cells/well in poly-l-lysine-coated, black µ-clear F-bottomed 96-well plates (Greiner) prefilled with warm plating medium.

Primary cells were left to settle for 1.5 h in a tissue culture incubator (37 °C, 5% CO_2_), and afterward the plating medium was changed to warm Neurobasal medium supplemented with 2% (vol/vol) B27, 0.25% (vol/vol) GlutaMAX, and 100 U/mL of penicillin–streptomycin. Primary cells were typically used in the toxicity assays at the age of 10 d in vitro.

### Immunofluorescence.

Primary neuronal cultures grown in 96-well plates were fixed in 3.6% (wt/vol) phosphate-buffered formaldehyde (100 µL/well) for 15 min, permeabilized for 5 min in 0.3% (vol/vol) TritonX100, 3% (wt/vol) bovine serum albumin (BSA) (Sigma), and 5% (vol/vol) horse serum (HS) (Thermo Fisher Scientific) in D-PBS (70 µL/well) and incubated with blocking buffer (3% [wt/vol] BSA and 5% [vol/vol] HS in D-PBS; 70 µL/well) for 1 h at room temperature. Anti-MAP2 mouse monoclonal antibody (1:400, Thermo Fisher Scientific, catalog no. M13-1500) and anti-spinophilin/neurabin-II rabbit polyclonal antibody (1:500, Millipore, catalog no. 06–852) were added to the cells to visualize neurites and dendritic spines, respectively. These antibodies were applied to cells in antibody buffer (3% [wt/vol] BSA, 1% [vol/vol] HS and 0.1% [vol/vol] Triton X100; 50 µL/well) and left overnight at 4 °C. Cells were then washed (2 × 100 µL D-PBS) and blocked (50 µL/well) with 5% (vol/vol) normal goat serum (Invitrogen) in D-PBS for 45 min. Next, the cells were incubated with secondary antibodies conjugated to Alexa Fluor dyes: goat anti-mouse Alexa647 (Thermo Fisher Scientific, catalog no. A21235) and goat anti-rabbit Alexa488 (Thermo Fisher Scientific, cat. No A11008) at a dilution of 1:1,000 in antibody buffer for 1.5 h at room temperature. Cells were then washed (2 × 100 µL D-PBS) and incubated with Alexa Fluor-555–conjugated anti-NeuN mouse antibody to visualize neuronal nuclei (1:500, Millipore, catalog no. MAB377A5, applied to cells in antibody buffer [50 µL/well] for 1 h at room temperature). The cells were washed again (2 × 100 µL D-PBS), and 150 µL of D-PBS was added to each well after the last wash. Then the plates were sealed and stored at 4 °C in the dark. Plates were imaged within 1 wk of staining.

### Image Acquisition.

Images were acquired either with an IncuCyte S3 (Sartorius) or Opera Phenix (Perkin-Elmer). For IncuCyte imaging, the imaging platform was kept inside a tissue culture incubator at 37 °C and 5% CO_2_. A total of four to nine images were taken per well with a 20× objective lens in phase contrast every 4 h from the same well coordinates. For Opera Phenix imaging, images of fixed, fluorescently stained cells were acquired with a 40× water immersion objective, and 15 views were taken per well. The laser power and exposure time was optimized for each channel. Flat-field profiles were calculated at imaging.

### Image Analysis.

Images taken on IncuCyte were analyzed with the NeuroTrack module, IncuCyte S3 v.2018B. Typical parameters used for the NeuroTrack analysis were as follows: segmentation mode—brightness; segmentation adjustment—1.2; neurite sensitivity—0.4; filtering—best; neurite width—2 µm. All images collected on the Opera Phenix were run through Acapella analysis scripts in Columbus (Perkin-Elmer). Flat-field correction was applied as the starting point for all analyses. Neuron counts were made by segmenting NeuN-positive nuclei using the “Find nuclei” function. Nuclear morphological measurements: area (µm^2^) and roundness (perimeter form factor [Ff] calculated from a function of perimeter and area [Ff for a circle = 1, other shapes < 1]) were calculated using the “Calculate morphological properties” function for each nucleus. Populations based on dimension were included and excluded from further analysis by the “Select population” function. Populations of nuclei smaller than 60 µm^2^ were designated as pyknotic. Populations of nuclei with roundness of under 0.65 Ff were designated as fragmented or blebbing. Neurons with nuclei that were classed as pyknotic or fragmented/blebbing were considered dead and those that were not classed as pyknotic or fragmented/blebbing were considered healthy. Neurite fragmentation was estimated from images by first identifying MAP2-postitive neurites using the “Find image region” function. This region was then split into distinct objects. The area of each MAP2 object was calculated in square micrometers, using the “Calculate morphology properties” function. Fragments were selected using the “Select population” function from the total object population if their area was less than or equal to 20 µm^2^. All objects over 20 µm^2^ were considered healthy neurites. The neurite fragmentation assay readout was the total number of fragments per image divided by the total number of neurons in the same image. Spinophilin-positive dendritic spines surrounding healthy neurites were counted using the “Find image region” and “Find surrounding region” functions in the spinophilin channel. Dendritic spines were then identified and segmented using the “Find spots: method A” function, solely within the two identified regions. The dendritic spine density assay readout was calculated as the number of spines per micrometer of healthy neurites.

### Statistical Analysis.

The average neurite length value from the four to nine images per well, acquired by IncuCyte, was found. There were at least three technical replicates per treatment tested in three independent experiments comprising independent cell cultures and independent prion preparations. Neurite length was normalized to the baseline neurite length recorded for ∼20 h prior to the treatment (100%) on a per-plate basis. For Columbus image analysis, analysis outputs of each image were averaged between 15 views of each technical replicate. Then, an average was found between all of the technical replicates from each independent test. For neurite fragmentation analysis, raw averages were normalized on a per-plate basis between 0 and 100% where 0% was the medium (negative control) and 100% was the 10% (wt/vol) RML BH diluted 100-fold to a concentration of 10^−3^. For dendritic spine density analysis, raw averages were normalized on a per-plate basis to obtain a percentage difference from the negative control. The mean and error of normalized values from all independent tests were plotted. Error bar type, statistical test, and *P* value are stated in figure legends. All statistical analyses were performed in GraphPad Prism 7.

## Supplementary Material

Supplementary File

Supplementary File

Supplementary File

Supplementary File

Supplementary File

Supplementary File

Supplementary File

## Data Availability

The data that support the findings of this study are available in the article and *SI Appendix*.

## References

[1] Prusiner S. B., Prions. Proc. Natl. Acad. Sci. U.S.A. 95, 13363–13383 (1998). 9811807 10.1073/pnas.95.23.13363 PMC33918

[2] Collinge J., Prion diseases of humans and animals: Their causes and molecular basis. Annu. Rev. Neurosci. 24, 519–550 (2001). 11283320 10.1146/annurev.neuro.24.1.519

[3] Collinge J., Clarke A. R., A general model of prion strains and their pathogenicity. Science 318, 930–936 (2007). 17991853 10.1126/science.1138718

[4] Collinge J., Mammalian prions and their wider relevance in neurodegenerative diseases. Nature 539, 217–226 (2016). 27830781 10.1038/nature20415

[5] Terry C., Wadsworth J. D. F., Recent advances in understanding mammalian prion structure: A mini review. Front. Mol. Neurosci. 12, 169 (2019). 31338021 10.3389/fnmol.2019.00169 PMC6629788

[6] Fang C., Imberdis T., Garza M. C., Wille H., Harris D. A., A neuronal culture system to detect prion synaptotoxicity. PLoS Pathog. 12, e1005623 (2016). 27227882 10.1371/journal.ppat.1005623 PMC4881977

[7] Fang C.., Prions activate a p38 MAPK synaptotoxic signaling pathway. PLoS Pathog. 14, e1007283 (2018). 30235355 10.1371/journal.ppat.1007283 PMC6147624

[8] Foliaki S. T.., Prion acute synaptotoxicity is largely driven by protease-resistant PrPSc species. PLoS Pathog. 14, e1007214 (2018). 30089152 10.1371/journal.ppat.1007214 PMC6101418

[9] Foliaki S. T.., Early existence and biochemical evolution characterise acutely synaptotoxic PrPSc. PLoS Pathog. 15, e1007712 (2019). 30970042 10.1371/journal.ppat.1007712 PMC6490942

[10] Goniotaki D.., Inhibition of group-I metabotropic glutamate receptors protects against prion toxicity. PLoS Pathog. 13, e1006733 (2017). 29176838 10.1371/journal.ppat.1006733 PMC5720820

[11] Meyer R. K.., Separation and properties of cellular and scrapie prion proteins. Proc. Natl. Acad. Sci. U.S.A. 83, 2310–2314 (1986). 3085093 10.1073/pnas.83.8.2310 PMC323286

[12] Cronier S.., Detection and characterization of proteinase K-sensitive disease-related prion protein with thermolysin. Biochem. J. 416, 297–305 (2008). 18684106 10.1042/BJ20081235 PMC2584334

[13] D’Castro L.., Isolation of proteinase K-sensitive prions using pronase E and phosphotungstic acid. PLoS One 5, e15679 (2010). 21187933 10.1371/journal.pone.0015679 PMC3004958

[14] Sandberg M. K.., Prion neuropathology follows the accumulation of alternate prion protein isoforms after infective titre has peaked. Nat. Commun. 5, 4347 (2014). 25005024 10.1038/ncomms5347 PMC4104459

[15] Sandberg M. K., Al-Doujaily H., Sharps B., Clarke A. R., Collinge J., Prion propagation and toxicity in vivo occur in two distinct mechanistic phases. Nature 470, 540–542 (2011). 21350487 10.1038/nature09768

[16] Frigg R., Klein M. A., Hegyi I., Zinkernagel R. M., Aguzzi A., Scrapie pathogenesis in subclinically infected B-cell-deficient mice. J. Virol. 73, 9584–9588 (1999). 10516067 10.1128/jvi.73.11.9584-9588.1999 PMC112993

[17] Hill A. F.., Species-barrier-independent prion replication in apparently resistant species. Proc. Natl. Acad. Sci. U.S.A. 97, 10248–10253 (2000). 10963685 10.1073/pnas.97.18.10248 PMC27848

[18] Asante E. A.., BSE prions propagate as either variant CJD-like or sporadic CJD-like prion strains in transgenic mice expressing human prion protein. EMBO J. 21, 6358–6366 (2002). 12456643 10.1093/emboj/cdf653 PMC136957

[19] Thackray A. M., Klein M. A., Aguzzi A., Bujdoso R., Chronic subclinical prion disease induced by low-dose inoculum. J. Virol. 76, 2510–2517 (2002). 11836429 10.1128/jvi.76.5.2510-2517.2002 PMC153817

[20] Wenborn A.., A novel and rapid method for obtaining high titre intact prion strains from mammalian brain. Sci. Rep. 5, 10062 (2015). 25950908 10.1038/srep10062 PMC4423448

[21] Terry C.., Ex vivo mammalian prions are formed of paired double helical prion protein fibrils. Open Biol. 6, 160035 (2016). 27249641 10.1098/rsob.160035 PMC4892434

[22] Terry C.., Structural features distinguishing infectious ex vivo mammalian prions from non-infectious fibrillar assemblies generated in vitro. Sci. Rep. 9, 376 (2019). 30675000 10.1038/s41598-018-36700-w PMC6344479

[23] Schmidt C.., A systematic investigation of production of synthetic prions from recombinant prion protein. Open Biol. 5, 150165 (2015). 26631378 10.1098/rsob.150165 PMC4703057

[24] Büeler H.., Normal development and behaviour of mice lacking the neuronal cell-surface PrP protein. Nature 356, 577–582 (1992). 1373228 10.1038/356577a0

[25] Büeler H.., Mice devoid of PrP are resistant to scrapie. Cell 73, 1339–1347 (1993). 8100741 10.1016/0092-8674(93)90360-3

[26] Prusiner S. B.., Scrapie prions aggregate to form amyloid-like birefringent rods. Cell 35, 349–358 (1983). 6418385 10.1016/0092-8674(83)90168-x

[27] Klöhn P. C., Stoltze L., Flechsig E., Enari M., Weissmann C., A quantitative, highly sensitive cell-based infectivity assay for mouse scrapie prions. Proc. Natl. Acad. Sci. U.S.A. 100, 11666–11671 (2003). 14504404 10.1073/pnas.1834432100 PMC208815

