# Supplementary material for: Highly infectious prions are not directly neurotoxic
Source: Proc Natl Acad Sci U S A. 2020 Sep 8;117(38):23815–22. doi: 10.1073/pnas.2007406117 (PMC7525444; doi:10.1073/pnas.2007406117)
Supplement: Supplementary File [file pnas.2007406117.sapp.pdf]

PNAS
www.pnas.org

Supplementary Information for

Highly infectious prions are not directly neurotoxic

Iryna Benilova[1, #], Madeleine Reilly[1, #], Cassandra Terry[1, &], Adam Wenborn[1], Christian Schmidt[1], Aline T. Marinho[1], Emmanuel Risse[1], Huda Al-Doujaily[1], Michael Wiggins De Oliveira[1], Malin K. Sandberg[1], Jonathan D.F. Wadsworth[1], Parmjit S. Jat[1, %], John Collinge[1, %, *]

[1]MRC Prion Unit at UCL, UCL Institute of Prion Diseases, Courtauld Building, 33 Cleveland Street, London W1W 7FF

[&]Present address: Molecular Systems for Health Research Group, School of Human Sciences, London Metropolitan University, London N7 8DB

[#]These authors contributed equally to this work

[%]Joint senior authors

[*]Correspondence and requests of materials should be addressed to J.C.

Email: jc@prion.ucl.ac.uk

This PDF file includes:

Figures S1 to S3
Legends for Movies S1 to S6

Other supplementary materials for this manuscript include the following:

Movies S1 to S6

www.pnas.org/cgi/doi/10.1073/pnas.2007406117

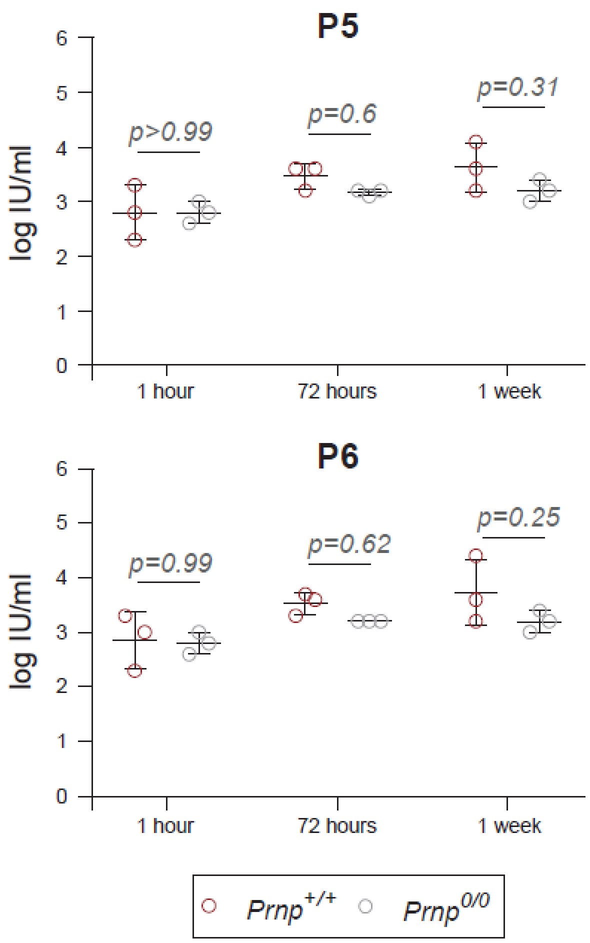

Fig. S1. Primary neuronal cultures do not propagate RML prions after a short treatment with RML prion-infected brain homogenate (RML BH). Three independent cortical cultures from *Prnp*$^{+/+}$ and *Prnp*$^{0/0}$ mice were treated with RML BH (brain concentration 10^{-4}) for 1 h, 72 h or 1 week. Infectivity titer was determined by Automatic Scrapie Cell Assay in PK1/2 cells after the 5th (P5) and 6th (P6) split, mean ± S.D. Infectivity in *Prnp*$^{0/0}$ cells represents residual infectivity of the inoculum. P values from 2-way ANOVA with Sidak's correction for multiple comparisons reveal no significant difference between genotypes upon any duration of treatment.

.

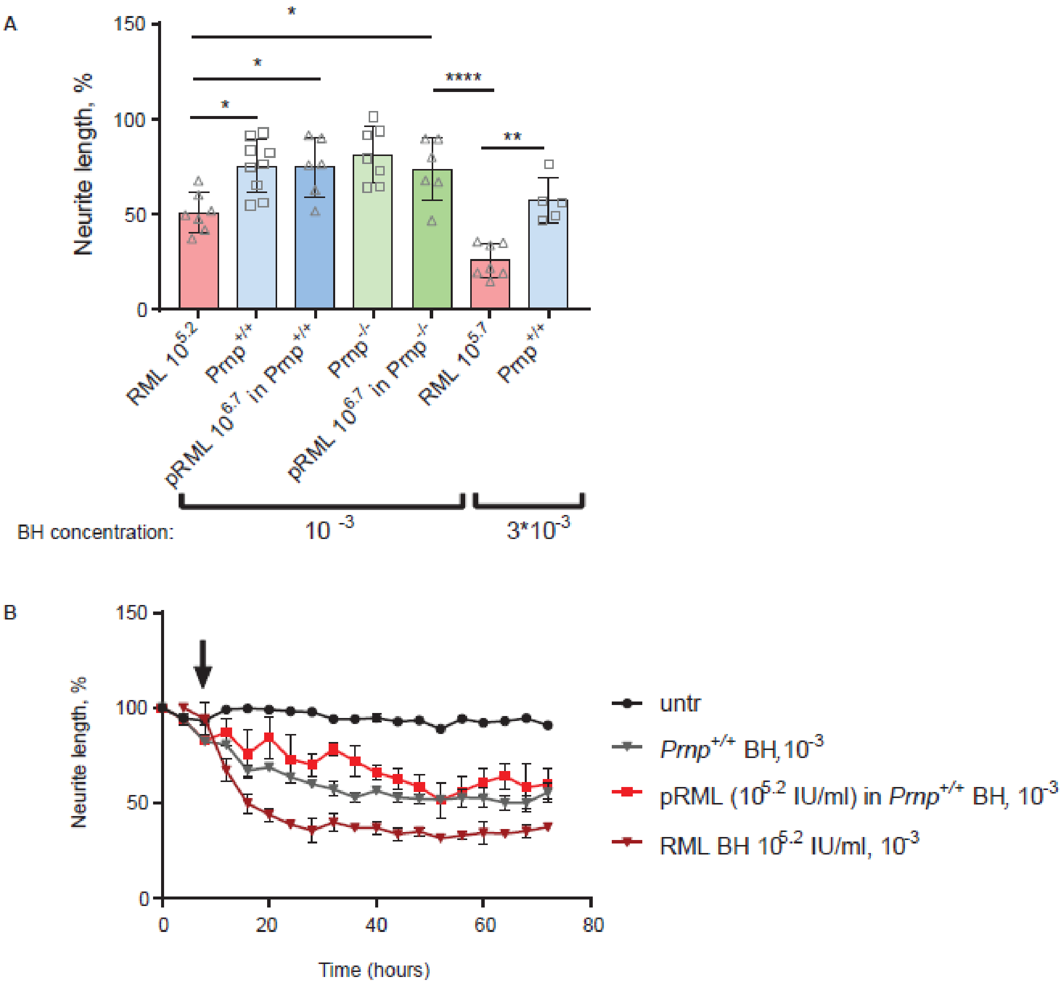

Fig. S2. Purified RML prions at $10^{6.7}$ IU/ml in *Prnp*$^{+/+}$ and *Prnp*$^{0/0}$ mouse brain homogenates at a brain concentration of 10^{-3} are not neurotoxic to primary neurons. **A**, Normalized response at 3 days post-treatment (n=5-9, mean ± S.D., NeuroTrack analysis parameters: sensitivity 0.4; neurite width: 1 µm). **B**, Normalized response over 68 h of treatment of primary neurons with pRML at $10^{5.2}$ IU/ml in *Prnp*$^{+/+}$ BH at 10^{-3} (n=3), RML BH at a concentration of 10^{-3} ($10^{5.2}$ IU/ml) (n=3) and uninfected *Prnp*$^{+/+}$ BH at 10^{-3} (n=3), mean ± S.E.M.

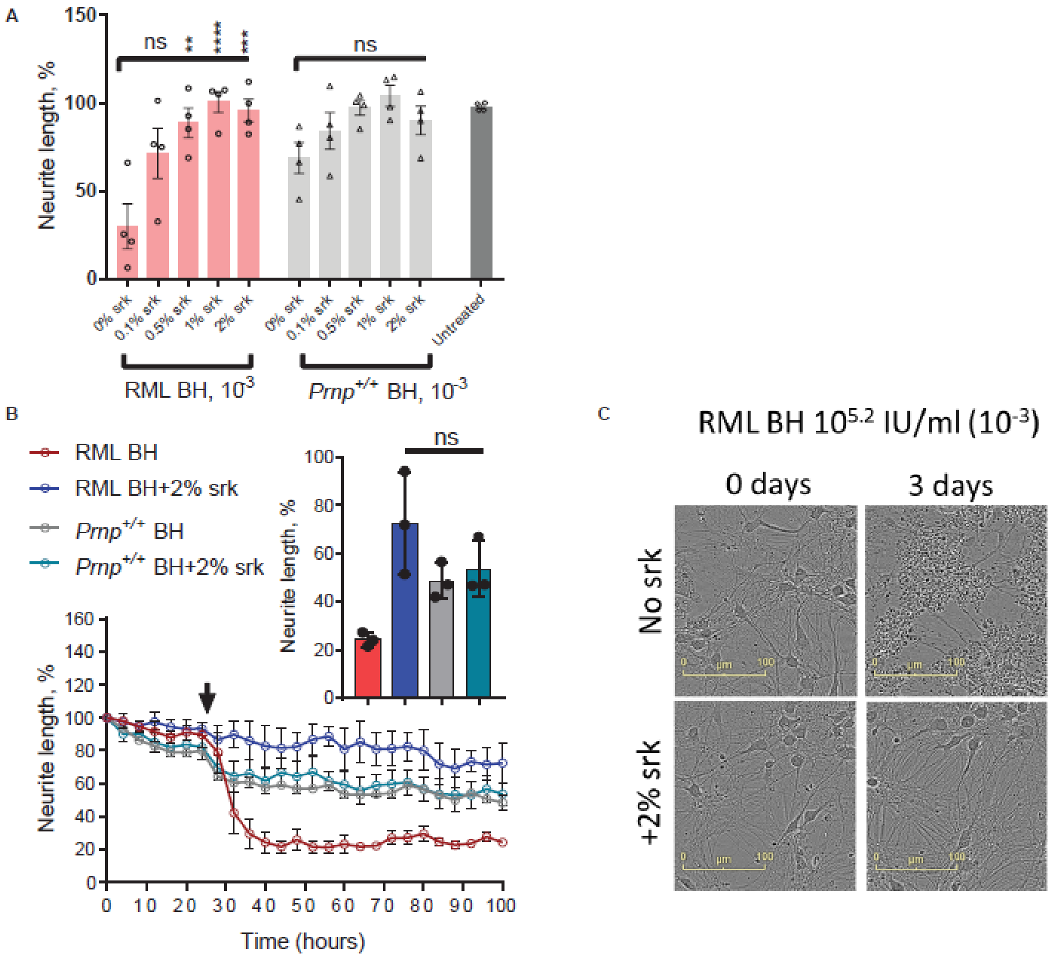

Fig. S3. Sarkosyl does not affect the non-specific toxicity of uninfected *Prnp*$^{+/+}$ brain homogenate. **A**, Normalized response at 12 h post-treatment (n=4, mean ± S.E.M., **$p<0.01$, ***$p<0.001$, ****$p<0.0001$, "ns": not significant, 1-way ANOVA with Dunnett's multiple comparisons test), **B** – Normalized response over 3 days of treatment with 2% (w/v) sarkosyl-pretreated *Prnp*$^{+/+}$ and RML BH at a brain concentration of 10^{-3} (n=3, mean ± S.E.M.) This dataset is independent of panel **A** and **Fig.5**. **B inset**, normalized response at 3 days post-treatment (n=3, mean ± S.D., "ns": not significant in an unpaired two-tailed t-test). **C**, Primary neurons look healthy after 3 days of treatment with 2% (w/v) sarkosyl-pretreated *Prnp*$^{+/+}$ BH at 10^{-3}, scale bar: 100 μm.

Movie S1 (separate file) Infectious RML brain homogenate (BH) has a pronounced toxic effect on neuronal network. RML BH containing $10^{4.2}$ infectious units (IU)/ml was added to 10 days old cortico-hippocampal mouse neurons after 20 hours of baseline recording. Note acute neurite retraction and loss of neuronal cell bodies.

Movie S2 (separate file) 10 days old neuronal culture treated with a non-infectious *Prnp*$^{+/+}$ brain diluted to a concentration of 10^{-4} (vehicle control for Movie S1). This brain homogenate was added to primary cells after 20 hours of baseline recording.

Movie S3 (separate file) High titre purified RML prions (infectivity $10^{6.7}$ IU/ml) diluted in tissue culture medium are not toxic to primary neurons. Prions were added to 10 days old cortico-hippocampal mouse culture after 20 hours of baseline recording.

Movie S4 (separate file) Non-infectious *Prnp*$^{0/0}$ brain diluted to a concentration of 10^{-4} reconstituted with high titre purified RML prions ($10^{6.7}$ IU/ml) is not toxic to primary neurons. Prions in brain homogenate were added to 10 days old cortico-hippocampal mouse neurons after 20 hours of baseline recording.

Movie S5 (separate file) 10 days old neuronal culture treated with a non-infectious *Prnp*$^{0/0}$ brain at a concentration of 10^{-4} (vehicle control for Movie S4). This brain homogenate was added to primary cells after 20 hours of baseline recording.

Movie S6 (separate file) Non-infectious *Prnp*$^{+/+}$ brain diluted to a concentration of 10^{-4} reconstituted with high titre purified RML prions ($10^{6.7}$ IU/ml) is not toxic to primary neurons. Prions were added to 10 days old cortico-hippocampal mouse neurons after 20 hours of baseline recording.
